# Pharmacovigilance of Risankizumab in the Treatment of Psoriasis and Arthritic Psoriasis: Real-World Data from EudraVigilance Database

**DOI:** 10.3390/pharmaceutics15071933

**Published:** 2023-07-11

**Authors:** Fabrizio Calapai, Ilaria Ammendolia, Luigi Cardia, Mariaconcetta Currò, Gioacchino Calapai, Emanuela Esposito, Carmen Mannucci

**Affiliations:** 1 Department of Clinical and Experimental Medicine, University of Messina, 98125 Messina, Italy; 2 Department of Chemical, Biological, Pharmaceutical and Environmental Sciences, University of Messina, 98125 Messina, Italy; 3 Department of Human Pathology of Adult and Childhood “Gaetano Barresi”, University of Messina, 98125 Messina, Italy; 4 Department of Biomedical and Dental Sciences and Morphological and Functional Imaging, University of Messina, 98125 Messina, Italy

**Keywords:** risankizumab, anti-IL-23, adverse reactions, pharmacovigilance, psoriasis, gender

## Abstract

Risankizumab is a selective, humanized immunoglobulin G1 (IgG1) monoclonal anti-body directed against interleukin (IL)-23 protein. The therapeutic indication of risankizumab is moderate-to-severe plaque psoriasis and psoriatic arthritis. The safety profile of risankizumab is currently defined by data obtained with clinical trials used for the authorization of entry into the market. The aim of this study was to expand information on the safety of risankizumab through a descriptive post-marketing analysis of real-world data regarding serious adverse reactions (SARs) to risankizumab found in the EudraVigilance database. The EudraVigilance database system, containing SARs linked to drugs not yet licensed for the market in the European Union (EU), was used. In EudraVigilance, SARs are described in single individual cases safety reports (ICSRs). More frequently reported serious SARs to risankizumab are associated with, in descending order, infections, cancer, nervous system disorders, cardiac disorders, abnormal laboratory results, pulmonary disorders, conditions aggravated, and skin disorders. Despite the classical limitations of this post-marketing study (lack of denominator, no certainty of causal relationship between the drug and the adverse reaction), analysis of real-world data related to SARs to risankizumab confirms the known safety profile of the drug but, at the same time, stimulates to further go into detail about the occurrence as adverse reactions of malignancies and their sex distribution.

## 1. Introduction

Psoriasis is a chronic, immune-mediated skin disease, and plaque psoriasis is the most common variant, accounting for more than 80% of psoriasis cases [1,2]. Plaque psoriasis is characterized by erythematous scaly patches. Psoriasis affects men and women equally and adults more than children [3,4]. Psoriatic arthritis usually arises approximately 10 years after the onset of psoriatic lesions [5] and clinically presents with dactylitis, as well as enthesitis of the plantar fascia and Achilles tendon [6]. Risankizumab is a humanized immunoglobulin G1 (IgG1) monoclonal antibody, produced using recombinant DNA technology and directed against interleukin (IL)-23 protein [7]. Risankizumab selectively binds with a high affinity to the p19 subunit of human cytokine IL-23, involved in inflammatory and immune responses, without binding to IL-12 and inhibits its interaction with the IL-23 receptor complex. By blocking IL-23 from binding to its receptor, risankizumab inhibits IL-23-dependent cell signaling and the release of proinflammatory cytokines [8]. The efficacy and safety of risankizumab have been investigated in people with moderate-to-severe plaque psoriasis [9,10,11]. The therapeutic indication of Risankizumab is “moderate to severe Plaque Psoriasis in adults who are candidates for systemic therapy and Psoriatic Arthritis alone or in combination with methotrexate”. Risankizumab is also indicated for the “treatment of moderate to severe plaque psoriasis in adults who are candidates for systemic therapy who have had an inadequate response or who have been intolerant to one or more disease-modifying antirheumatic drugs (DMARDs)” (EMA). Together with other IL-23 inhibitors, guselkumab and tildrakizumab, risankizumab to the same class of biologic drugs that have been licensed for the therapy of moderate-to-severe psoriasis [12].

The safety profile of risankizumab is currently defined by data obtained with clinical trials used for market authorization. A retrospective cohort study comparing indirectly the effectiveness and safety of risankizumab and guselkumab detected no cases of serious adverse events, neither injection site reaction, candida, cancer, cardiovascular reactions in both groups [13]. Another real-life study, performed by the same authors, confirmed that risankizumab is an effective and safe treatment option in routine dermatological practice. However, the authors concluded that more studies are needed to confirm the actual available safety data [14]. Another review asserted that anti-IL-23 agents for patients with psoriasis, including risankizumab, are generally well-tolerated and have high safety profiles. In this case, data were collected from phase III clinical trials and not from clinical practice [15]. A retrospective observational multicenter real world study, investigating on people with moderate-to-severe plaque psoriasis treated with risankizumab, was conducted with the aim to assess the effectiveness and safety of risankizumab in the medium-term (52 weeks). This retrospective analysis led the authors to conclude that risankizumab shows a high effectiveness profile in real clinical practice [16]. Since the current risk profile of risankizumab is essentially derived from pre-registration clinical studies, it is innovative and necessary to evaluate it through the analysis of real-world data. With the aim of making a contribution towards the definition of a safety profile and to expand information on the safety of risankizumab, beyond the most frequent adverse effects highlighted through clinical studies conducted prior to authorization, a descriptive analysis of data from real-life, generated through the database EudraVigilance, on suspected adverse reactions (SARs) to the use of this drug in patients affected by psoriasis was performed [17]. In this article were analyzed real world data on SARs to risankizumab collected by the database EudraVigilance, a database collecting SARs to drugs authorized for the market in the European Union (EU). The European Medicines Agency (EMA) is responsible for EudraVigilance, a system accumulating reports of SARs originating from national regulatory authorities and drug companies.

## 2. Methods

EudraVigilance is a database reporting SARs related to drugs yet authorized for the market or currently investigated in clinical trials in the European Union (EU). In this data bank, as happens for the other drugs, SARs to risankizumab are described in single individual cases safety reports (ICSRs). The information on this website relates to suspected adverse reactions to medical events that have been observed following the use of a medicine, but which are not necessarily related to or caused by the medicine [18]. Licensed drugs based on risankizumab as an active principle received a marketing authorization valid throughout the EU on 26 April 2019 [19]. The EudraVigilance data system contains all ICSRs reported by healthcare professionals or non-healthcare professionals to own EU national competent authorities or to marketing authorization holders. For our analysis, only ICSRs reporting SARs to risankizumab signaled by healthcare professionals were retrieved from 1 January 2020 to 31 December 2022. ICSRs are originating from European Economic Area (EEA) countries or not. In the present work, only ICSRs coming from EEA countries and United Kingdom (UK) were taken in consideration for the analysis. Public version of EudraVigilance database [20] has been used. ICSRs reporting SARs were selected on the basis of Medical Dictionary tor Regulatory Activities (MedDRA) [21]. MedDRA is a standardized international medical terminology used by regulatory authorities and pharmaceutical companies. It is used for coding cases of adverse effects in clinical study safety reports and pharmacovigilance databases [22]. Information on patient’s age and sex, type of adverse events (often more than one for each ICSR), source, and drugs taken concomitantly, were extracted from all ICSRs. Only serious cases analyzed. In accordance with the International Council on Harmonization E2D guidelines, ICSRs are classified as serious if it is life-threatening, resulted in death, caused/prolonged hospitalization or disability, related to a congenital anomaly/birth defect or other medically important condition. Duplicate and incomplete ICSRs were considered as missing data/not reported and excluded from the analysis. Some details are not always available in EudraVigilance and it is likewise important to take in consideration that restrictions of the database to obtain subgroup analysis may produce other biases potentially causing the under-detection of signals that would be detected if filters to the whole dataset are not used. The characteristics and limits fixed to collect ICSRs related to risankizumab from EudraVigilance were only serious SARs, complete for age and sex information, indication, and concomitant drugs. A descriptive statistical analysis of data was carried out in the present study by using the statistical software SPSS version 28.0.0.0 (190). The appropriate stratification of signals by groups for age and sex was used to reduce bias due to confounding effects due to these variables. The reporting odds ratio (ROR), a disproportional signal detection method, was employed to detect the association between cancer as a suspect adverse reaction and risankizumab, by calculating ROR with its 95% confidence interval (CI) for risankizumab, using as the controls SARs to substances belonging to the same class of drugs (IL-23 inhibitors), guselkumab and tildrakizumab, used for the treatment of psoriasis [23].

## 3. Results

ICSRs reporting risankizumab as a drug involved in SARs, during the three years of the period January 2020–December 2022 (years 2020-2021-2022), were retrieved from EudraVigilance (accessed on 31 December 2022). Currently, the total number of ICSRs risankizumab-related of both serious and non-serious SARs in EEA countries and the UK in EudraVigilance for the years 2020, 2021, and 2022 is 855: 103/125 (2020 = 228), 116/169 (2021 = 285), and 148/194 (2022 = 342) (checked on 31 December 2022). The number of serious ICSRs of the years 2020, 2021, and 2022 is 141 (16.5%) of the total number of ICSRs related to risankizumab in EudraVigilance database in the same three years. ICSRs not complete for age or sex or both were excluded by the descriptive analysis. Consequently, analysis was performed on 110 serious ICSRs and compared with other serious SARs found in the database. Classification for age group, calculated on serious ICSRs, indicates that the age most affected was that represented by adults aged 18–64 years (78.2%), while the remaining ICSRs are focused on people aged 65–85 years (21.8%). In EudraVigilance, ICSRs with serious SARs to risankizumab in men are present in the largest number with respect to women (60.9 vs. 39.1%). Part of SARs involves the use of risankizumab prescribed with other drugs, but most serious ICSRs are related to the use of risankizumab alone (61.9%). Death was detected in nine of the serious cases (8.2%) (Table 1). Among the drugs prescribed with risankizumab, methotrexate is the only one indicated to be associated with the authorization market. Results of the analysis show that in ICSRs reporting serious SARs to risankizumab, this drug was prescribed in 8.2% of cases, while the first drug to be prescribed (11.8%) in association with risankizumab is adalimumab, a drug indicated for the treatment of active psoriatic arthritis in adults [24].

Listed in Table 2 are the SARs more frequently reported in serious risankizumab-related ICSRs (Table 2). Serious SARs more frequently reported are associated with, in descending order, infections, cancer, nervous system disorders, and cardiac disorders, followed by abnormal laboratory results, pulmonary disorders, conditions aggravated, and skin disorders. Reported in Table 2 are the groups of SARs collected for risankizumab according to age stratification while in Table 3 are showed serious SARs according to sex distribution. In Table 4, SARs to risankizumab are reported according to reaction groups.

ROR calculated between data on cancer as a serious adverse event suspected to be caused by risankizumab and the serious SARs of other drugs of the same class gave a result of 1.37, considered significant when the lower limit of the 95% confidence interval is greater than 1 (Table 5).

## 4. Discussion

IL-23 inhibitors are the newest class of biologic drugs used for the therapy of psoriasis. Pre-registration clinical studies conducted with these three drugs produced favourable results [12]. Despite the great importance of clinical trials, real-life studies are considered identically important due to the involvement of a different cohort of patients often suffering from multiple comorbidities, as well as subjects affected by other forms than plaque psoriasis. These patients are generally excluded by the tight inclusion and exclusion criteria guiding the recruitment of clinical trials. Furthermore, data from real-world settings may include patients affected by infections such as latent tuberculosis, as well as patients with other pathologies [25]. Consequently, real-world studies represent an important source of data useful to improve the decision-making progress, guiding medical doctors to choose the best individual therapy.

In this context, real-world data about IL-23 inhibitors appear to confirm the favourable results of clinical trials, highlighting the efficacy and safety profiles of this class of biologic drugs also in the routinary clinical practice. One of these drugs, risankizumab, has been proved to be an effective and safe treatment option in routine dermatological practice, achieving a very significant improvement in the quality of life of patients and showing excellent outcomes even in more fragile patients suffering from multiple comorbidities [26,27].

According to the EMA risk management plan associated with market authorization, potential important risks related to risankizumab prescription are major cardiac events, serious infections, malignancies, and serious hypersensitivity reactions. In this regard, an incidence of 12–15% of serious adverse events linked to risankizumab use has been reported, including two basal cell carcinomas and a major cardiovascular event [28], while other clinical trials have reported a lower percentage of severe adverse events ranging between 1.67 and 5.5% [29]. Adverse events related to risankizumab most frequently reported trough clinical trials are upper respiratory tract infections, nasopharyngitis, headache, and arthralgia [9,30,31]. Real-world studies on risankizumab safety show contradictory outcomes, some of them reporting no safety findings beyond those reported in the pre-registration trials, while, more recently, the increased reporting of cerebrovascular accidents with the use of risankizumab has been observed through the analysis of the database of spontaneous reporting signals of the Food and Drug Administration Adverse Events Reporting System (FAERS) [32] (Table 6). Another study tried to assess the safety of risankizumab by the analysis of real-world sample pharmacovigilance database through the quantification of 10,235 reports of risankizumab-related adverse events collected from the second quarter of 2019 to third quarter of 2022. Results of this analysis indicated that severe adverse events, including pneumonia, cerebrovascular accidents, myocardial disorders, and liver cirrhosis, should be highly concerned [33].

On this basis, the aim of the study was to obtain post-marketing real-world data on the safety of risankizumab used for the therapy of psoriasis and arthritic psoriasis through the analysis of spontaneous pharmacovigilance signals findable in the European EudraVigilance database.

Descriptive analysis of the present study reveals that in EEA countries and the UK, the rate of serious adverse events to risankizumab is 16.5% with respect to the total number of SARs (serious and non-serious) signaled in EudraVigilance for this drug. It also reveals that serious SARs to risankizumab occur most frequently among men and when the drug is prescribed alone. A fatal outcome was reported in 8.2% of serious cases signaled in EudraVigilance (Table 1). Most common SARs resulted in being infections, cancer, nervous system disorders, or cardiac disorders (Table 2).

As reported in the Summary of Product Characteristics released by EMA, risankizumab may increase the risk of infections. In the same document, EMA recommends that risankizumab should be used with caution in patients with a chronic infection, a history of recurrent infection, or known risk factors for infection. Moreover, it also clarifies that therapy with risankizumab should not be initiated in patients with any clinically important active infection until the infection resolves or is adequately treated [34]. This recommendation raises the knowledge that risankizumab may reduce immune function, as happens with many immune-modulating agents, thus producing an increase in the risk of serious infection [35]. Real-world data of the present study seem to confirm, through the analysis of signals deriving from SARs, that the occurrence of infections can be a serious adverse event associated with risankizumab prescription. At the same time, it can be observed that for the most part, these are infections of the respiratory tract. Moreover, cases found in EudraVigilance are most related to COVID-19 infections and they can be explained by the fact that the years 2020-2021-2022, taken into consideration for this descriptive analysis, partially coincided with the peak of the COVID-19 pandemic. The occurrence of infections as potential adverse events to risankizumab was investigated in several clinical studies. In two 24-week clinical studies collecting data from the short-term treatment of psoriatic arthritis with risankizumab, serious infections were reported for 1.0% and 1.2% of patients receiving the drug and placebo, respectively [36], and 0.9% and 2.3% of patients receiving risankizumab and placebo, respectively [37]. In a long-term 52-week study on risankizumab use for psoriatic arthritis, carried out by the same group of researchers, the rate of serious infections was consistent with the previously observed week 24 rate [38]. Present real-world data extracted from EudraVigilance show that infections are 20.9% of the total number of SARs related to risankizumab prescription, without any significant gender difference or any difference between adult or elder people.

Treatment with risankizumab also requires attention to the presence of tuberculosis infection. Psoriasis is an immune-mediated, inflammatory disease, and it is well known that the tumor necrosis factor (TNF)-α inhibitors are associated with enhanced risk of reactivation of latent tuberculosis infection. Indeed, international guidelines suggest evaluating patients for tuberculosis infection before initiating therapy with the IL-23 inhibitor risankizumab, considering caution regarding the application of anti-tuberculosis treatment in patients with a history of this latent or active infection. This happened because the prescription of IL-23 inhibitors for the treatment of psoriasis renewed the problem of potential tuberculosis reactivation, based on the possibility that IL-23 antagonism does carry the same risk of tuberculosis reactivation as TNF-α inhibitors do, based on preclinical studies that have indicated that IL-23 has a potential role against tuberculosis infection [39]. However, it has been observed that after risankizumab therapy for 55 weeks, no active tuberculosis developed, despite the lack of prophylaxis, in a small number of patients with latent tuberculosis that did not receive tuberculous prophylaxis before treatment [11]. In EudraVigilance database, one reaction characterized by skin rash was reported in a person with latent tuberculosis, but no signs of manifest infection were signaled. Furthermore, to date, there is no evidence of reported cases of tuberculosis reactivation under risankizumab in real-world settings [29].

In EudraVigilance database, after infections, in order of frequency, are malignancies associated with SARs to risankizumab. They are mostly solid tumors such as lung cancer, squamous cell carcinoma, prostate cancer with two hematologic tumors (diffuse large B-cell lymphoma and IgA myeloma), and other variously distributed tumors. It is well known that psoriatic patients may have higher rates of neoplastic diseases than the general population, regardless of treatment, and equilibrium between cytokines IL-23 and IL-12 can affect antitumor and pro-tumor immune activities [40]. Moreover, patients affected by psoriasis are at enhanced risk of developing non-melanoma skin cancer (NMSC), especially those who received early systemic therapy or phototherapy [41].

Chronic inflammatory state, typical of psoriasis, may induce pro-tumorigenic effects; however, the discussion of the potential risk of malignancy in patients taking these drugs remains largely unsolved [42]. Data from the present descriptive analysis show three cases of skin cancer signaled as SARs related to risankizumab, two associated with squamous cell carcinoma and one with melanoma. But the most interesting data concern the gender distribution of SARs related to risankizumab associated with cancer. It can be observed that in EudraVigilance database, most of the suspected cases related to risankizumab prescription concern prevalently men, representing 77.8% of SARs associated with cancer. Even though the increased risk of patients affected by psoriasis for cancer has been detected, and evidence suggests that they might have a higher risk of skin cancer, especially for NMSC, compared with psoriasis-free patients [43], the reasons for this increased risk remain to be determined, and association with other cancers or gender difference has not yet been revealed.

A recent analysis of long-term phase I–III clinical trials (17 studies), conducted to evaluate safety data from risankizumab treatment for psoriasis, suggested that its use does not increase the risk of malignancies over that already associated with the pathophysiology of psoriasis but contemporarily suggested that patients should be evaluated for risk of malignancies both before and during treatment. In this analysis of long-term studies with risankizumab, no lymphoma or hematological malignancy were reported, whereas basal cell carcinoma and skin squamous cell carcinoma were observed in 23 and 14 patients, respectively [44].

The disproportionality analysis used in EudraVigilance is the reporting odds ratio (ROR). ROR is a proportion of cases for a drug–adverse event association in relation to the proportion of cases that would be expected if no combination existed between the drug and the adverse event. ROR use is based on the hypothesis that when a product causes an event, the number of signaled reports for the association will tend to be greater than that based on chance alone [45]. Our disproportionality analysis shows a link between the occurrence of cancer and use of risankizumab in the therapy of psoriasis. Similar results were observed by other authors who found a significant signal for risankizumab associated with benign, malignant, and unspecified neoplasms (ROR = 1.19). In light of these results obtained by the data extracted by EudraVigilance, it becomes necessary to further investigate the origin of the gender distribution of cancer, suspected to be related with risankizumab prescription.

The occurrence of cardiac disorders represents another significant concern about serious SARs related to risankizumab. This typology of risk is yet known, and increased risk for myocardial infarction, cardiovascular mortality, together with the enhancement of risk for stroke, has been reported in patients with psoriasis and psoriatic arthritis [46,47]. Two meta-analyses showed that patients with psoriasis and psoriatic arthritis have a 29% and 55% higher risk of developing myocardial infarction, respectively [48,49]. This increased risk has been attributed not only to a higher prevalence of traditional vulnerable factors (hypertension, obesity, diabetes, and hyperlipidemia) but also as a result of chronic systemic inflammation related to the skin developing these diseases [50]. Our descriptive analysis reveals that cardiac disorders are 10.9% of serious SARs to risankizumab, with no sensible gender difference. The SAR more frequent in this group is myocardial infarction (Table 4), being signaled eight times out of the total number of thirteen serious cardiac disorders reported as SARs in ICSRs.

The entry of biological treatment for psoriasis created the expectation of control over cardiovascular comorbidity [51], and results from retrospective studies have supported the hypothesis that biological drugs may purchase myocardial protection, reducing the probability that patients with psoriasis could develop cardiovascular diseases [52,53]. However, the role of these drugs in vascular damage processes remains controversial, probably due to the inconsistency of clinical data on their efficacy against increased cardiovascular risk [54]. These new data extracted from EudraVigilance database, about suspected cardiac disorders related to risankizumab prescription, are currently not easily explained. About this, a retrospective disproportionality analysis was conducted utilizing post-marketing adverse event reports submitted to the United States Food and Drug Administration Adverse Event Reporting System (FAERS) through the fourth quarter of 2021. This study, based on FAERS real-world data, showed the association of risankizumab with significantly disproportionate cerebrovascular accident reporting in comparison with all the other drugs in the system [55]. Finally, other serious SARs, which can be included in the reaction groups characterized by nervous system disorders, were found to be potentially related to risankizumab but without producing any particular alarm signs. They were neurologic disorders, such as two cases of epilepsy, two cases of aggravation of schizophrenia, and single cases of polyradiculopathy, polyneuropathy, motor ataxia, memory loss, coma, obnubilation, cerebrovascular accident, and depression.

**Table 6:** Severe adverse reactions to risankizumab from real-world studies.

Title of the Study	Serious Adverse Events	Reference
Real-world practice indirect comparison between guselkumab and risankizumab: Results from an Italian retrospective study.	No serious adverse events, injection site reaction, candida, cancer, cardiovascular events were reported.	[13]
Risankizumab: Efficacy, Safety, and Survival in the Mid-Term (52 Weeks) in Real Clinical Practice in Andalusia, Spain, According to the Therapeutic Goals of the Spanish Psoriatic Guidelines.	Risankizumab does not show safety findings beyond those previously reported in the pivotal clinical trials.	[16]
Increased reporting of cerebrovascular accidents with use of risankizumab observed in the Food and Drug Administration Adverse Events Reporting System (FAERS).	An increase in reporting of cerebrovascular accidents was detected following use of risankizumab for psoriasis.	[32]
Adverse events with risankizumab in the real world: postmarketing pharmacovigilance assessment of the FDA adverse event reporting system.	Pneumonia, cerebrovascular accident, myocardial infarction, cardiac disorder, and hepatic cirrhosis.	[33]
Potential cerebrovascular accident signal for risankizumab: A disproportionality analysis of the FDA Adverse Event Reporting System (FAERS).	This study identified several potential cerebrovascular accidents as adverse reactions signals for risankizumab.	[55]

Present real-life data, generated through the database EudraVigilance, on SARs to risankizumab in patients affected by psoriasis generally confirms the risk profile of pre-registration studies, except for some issues. The risk profile designed with pre-registration clinical studies is confirmed by the prevalence of infections, cancer, nervous system disorders, cardiac disorders, abnormal laboratory results, pulmonary disorders, conditions aggravated, and skin disorders, as more SARs signaled. The outcomes of the study suggest the existence of asymmetrical sex distribution in the occurrence of serious adverse effects, showing that men are more affected. Most of the SARs in EudraVigilance seem to be caused by risankizumab with a minor concern about potential drug interactions. A separate issue is represented by the difference in cancer signaling and its sex distribution, with men being more affected, something that deserves to be investigated more thoroughly. On this basis, the present study contributes to improving the definition of the safety profile by expanding the information which has been, until now, mostly limited to the most frequent adverse effects highlighted through clinical studies conducted prior to authorization. The limits of the present study are typical of large databases containing real-world data, which have their strength in terms of the quantity of reports and weakness due to lying about the quality of the single report. In addition, the database contains the signaling of SARs, meaning any adverse event for which there is a reasonable possibility, the suspicion, but not the certainty, that the drug is the cause of the reaction. Moreover, disproportionality analysis performed to obtain ROR only provides an evaluation of the signal strength. Due to the lack of a denominator and to the under-reporting phenomenon, ROR does not quantify the true clinical risk. Furthermore, in some cases, patients with psoriasis may take other drugs, influencing immune system activity, such as immunosuppressive agents, consequently impairing defense against neoplastic diseases. Even though we wish to reiterate these limits, descriptive analysis performed on EudraVigilance database shows some interesting aspects linked to risankizumab prescription for psoriasis and psoriatic arthritis. In conclusion, EudraVigilance data on risankizumab partially confirm the risk management plan of EMA, signaling the possibility of an increase in infections, malignancies, and cardiac disorders. However, the more interesting information originated by the analysis of SARs in EudraVigilance is certainly, together with the increase in suspected malignant tumors, its gender distribution, because in a asymmetric perspective, men seem to be more affected with respect to women. Unfortunately, the incidence rate cannot be calculated by spontaneous reports; thus, further experimental and prospective clinical studies are still needed to confirm the results. This analysis of real-world data related to SARs of risankizumab stimulates going into further detail about the possibility of the gender distribution of malignancies suspected to be linked to its prescription. For these reasons, also considering that risankizumab is getting ready to be more widely utilized in people with Crohn’s disease as well as psoriasis, in clinical practice, physicians and patients should be informed of these new, potentially severe, pharmacovigilance signals.

## Figures and Tables

**Table 1:** Serious suspected adverse reactions (SARs) related to the prescription of risankizumab in European Economic Area (EEA) according to sex, concomitant use of risankizumab with other drugs, and death, reported in individual case safety reports (ICSRs) in the years 2019–2022. Data are reported as a percentage of the total number of ICSRs (N = 110).

Sex reported in ICSRs with serious SARs to risankizumab	39.1%/60.9% (females/males)
Serious ICSRs reporting a concomitant use of risankizumab with other drugs	38.1%
Death reported as outcome of SARs in serious ICSRs	8.2%

**Table 2:** Percentage of individual case safety reports (ICSRs) signaling more frequent serious suspected adverse reactions (SARs) associated with risankizumab use in European Economic Area (EEA) and United Kingdom displayed in EudraVigilance for the years 2020-2021-2022 according to age distribution.

SARs	All the Ages (18–85 Years) % of Total ICSRs (N = 110)	% of Total Serious ICSRs in the Age Group of 18–64 Years (N = 86)	% of Total Serious ICSRs in the Age Group of 65–85 Years (N = 24)
Infections	20.0%	20.9%	16.7%
Cancer	16.4%	17.4%	12.5%
Cardiac disorders	10.9%	10.5%	12.0%
Nervous system disorders	10.9%	9.3%	16.7%

**Table 3:** Percentage of individual case safety reports (ICSRs) signaling more frequent serious suspected adverse reactions (SARs) associated with risankizumab use in European Economic Area (EEA) and United Kingdom displayed in EudraVigilance for the years 2010-2021-2022 according to sex.

SARs	Female Serious ICSRs within Each Reaction Group	Male Serious ICSRs within Each Reaction Group	Female Serious ICSRs in all the Cases (N = 110)	Male Serious ICSRs in all the Cases (N = 110)	Total Number of Cases for Each Reaction Group
Infections	45.4%	54.5%	9.1%	10.9%	22
Cancer	22.2%	77.8%	3.6%	12.7%	18
Cardiac disorders	53.8%	46.1%	5.4%	6.4%	13
Neurologic and psychiatric disorders	33.3%	66.7%	3.6%	7.3%	12

**Table 4:** Suspected adverse reactions (SARs) to risankizumab signaled in individual case safety reports (ICSRs) of EudraVigilance in European Economic Area (EEA) and United Kingdom during the years 2020-2021-2022 in the reaction groups of infections, cancer, cardiac disorders, and nervous system disorders. In brackets is the number of individual case safety reports (ICSRs) in which the reaction is reported.

Infections	Cancer	Neurologic and Psychiatric Disorders	Cardiac Disorders
Parotitis	Lung cancer (3)	Schizophrenia aggravated (2)	Myocardial infarction (8)
Viral infection	Squamous cell carcinoma (2)	Depression	Non-ST segment elevation
Fungal infection	Testicular cancer	Epilepsy (2)	Cardiac arrest
Interstitial pneumonitis (2)	Carcinoma larynx	Obnubilation	Not specified cardiac disorder (2)
COVID-19 (9)	IgA myeloma	Polyradiculopathy	Heart failure
Not specified respiratory tract infection	Diffuse Large B-cell lymphoma	Coma	
Herpetic keratitis	Melanoma	Polyneuropathy	
Cryptosporidiosis infection	Gastric carcinoma	Cerebrovascular accident	
Surgical wound infection	Cervical cancer	Loss of consciousness	
Borrelia infection	Oesophageal carcinoma	Motor ataxia	
Candida infection	Prostate cancer (2)	Memory loss	
Monkeypox	Carcinoma of tongue		
Lung abscess	Glioblastoma		
	Vulvar carcinoma		

**Table 5:** Reporting odds ratio (ROR) related to relevance of cancer as serious suspected adverse reaction to risankizumab vs. the drugs guselkumab and tildrakizumab. Data from EudraVigilance.

Drug	Cancer	All the Other Serious Adverse Reactions	ROR (95% CI)
Risankizumab	18	91	1.62 (0.87–3.01)
Guselkumab + tildrakizumab	34	279	

CI = confidence interval.

## Data Availability

Data analyzed for this study are available at the public website: https://www.ema.europa.eu/en/human-regulatory/research-development/pharmacovigilance/eudravigilance (accessed on 31 December 2022).

## References

[1] Michalek I.M., Loring B., John S.M. (2017). A systematic review of worldwide epidemiology of psoriasis. J. Eur. Acad. Dermatol. Venereol..

[2] World Health Organization (2016). Global Report on Psoriasis: World Health Organization. https://apps.who.int/iris/bitstream/handle/10665/204417/9789241565189_eng.pdf.psoriasis?sequence=1.

[3] Rachakonda T.D., Schupp C.W., Armstrong A.W. (2014). Psoriasis prevalence among adults in the United States. J. Am. Acad. Dermatol..

[4] Paller A.S., Singh R., Cloutier M., Gauthier-Loiselle M., Emond B., Guérin A., Ganguli A. (2018). Prevalence of Psoriasis in Children and Adolescents in the United States: A Claims-Based Analysis. J. Drugs Dermatol..

[5] Moll J.M., Wright V. (1973). Psoriatic arthritis. Semin. Arthritis Rheum..

[6] Ritchlin C.T., Colbert R.A., Gladman D.D. (2017). Psoriatic arthritis. N. Engl. J. Med..

[7] McDonald J., Maliyar K., Gooderham M.J. (2020). Risankizumab, an IL-23p19 Inhibitor for Psoriasis: A Review of the Current Literature. Ski. Ther. Lett..

[8] Fragoulis G.E., Siebert S. (2022). The role of IL-23 and the use of IL-23 inhibitors in psoriatic arthritis. Musculoskelet. Care.

[9] Ohtsuki M., Fujita H., Watanabe M., Suzaki K., Flack M., Huang X., Kitamura S., Valdes J., Igarashi A. (2019). Efficacy and safety of risankizumab in Japanese patients with moderate to severe plaque psoriasis: Results from the SustaIMM phase 2/3 trial. J. Dermatol..

[10] Lytvyn Y., Mufti A., Zaaroura H., Sachdeva M., Lu J.D., Rankin B.D., Prajapati V.H., Vender R., Yeung J. (2021). Efficacy and safety of risankizumab for moderate-to-severe plaque psoriasis in clinical practice: A 16-week Canadian retrospective multicenter cohort study. JAAD Int..

[11] Blauvelt A., Leonardi C.L., Gooderham M., Papp K.A., Philipp S., Wu J.J., Igarashi A., Flack M., Geng Z., Wu T. (2020). Efficacy and Safety of Continuous Risankizumab Therapy vs. Treatment Withdrawal in Patients with Moderate to Severe Plaque Psoriasis: A Phase 3 Randomized Clinical Trial. JAMA Dermatol..

[12] Ruggiero A., Picone V., Martora F., Fabbrocini G., Megna M. (2022). Guselkumab, Risankizumab, and Tildrakizumab in the Management of Psoriasis: A Review of the Real-World Evidence. Clin. Cosmet. Investig. Dermatol..

[13] Ruggiero A., Fabbrocini G., Cinelli E., Megna M. (2022). Real world practice indirect comparison between guselkumab and risankizumab: Results from an Italian retrospective study. Dermatol. Ther..

[14] Ruggiero A., Fabbrocini G., Cinelli E., Ocampo Garza S.S., Camela E., Megna M. (2022). Anti-interleukin-23 for psoriasis in elderly patients: Guselkumab, risankizumab and tildrakizumab in real-world practice. Clin. Exp. Dermatol..

[15] Naik P.P. (2022). Adverse Effects of Anti-Interleukin-23 Agents Employed in Patients with Psoriasis: A Systematic Review. Dermatology.

[16] Ruiz-Villaverde R., Rodriguez-Fernandez-Freire L., Pérez-Gil A., Font-Ugalde P., Galán-Gutiérrez M. (2022). Risankizumab: Efficacy, Safety, and Survival in the Mid-Term (52 Weeks) in Real Clinical Practice in Andalusia, Spain, According to the Therapeutic Goals of the Spanish Psoriatic Guidelines. Life.

[17] EudraVigilance. https://www.ema.europa.eu/en/human-regulatory/research-development/pharmacovigilance/eudravigilance.

[18] Ammendolia I., Mannucci C., Cardia L., Calapai G., Gangemi S., Esposito E., Calapai F. (2023). Pharmacovigilance on cannabidiol as an antiepileptic agent. Front. Pharmacol..

[19] Risankizumab Authorization Details. https://www.ema.europa.eu/en/medicines/human/EPAR/skyrizi#authorisation-details-section.

[20] EudraVigilance Database. http://www.adrreports.eu/en/search.html.

[21] (2022). Introductory Guide MedDRA Version 25.1. https://admin.meddra.org/sites/default/files/guidance/file/intguide_25_1_English.pdf.

[22] (2016). MedDRA and Pharmacovigilance: A Complex and Little Evaluated Tool. Prescrire Int..

[23] Vu A., Ulschmid C., Gordon K.B. (2022). Anti-IL 23 biologics for the treatment of plaque psoriasis. Expert Opin. Biol. Ther..

[24] Subramonian A., Peprah K., Picheca L. (2020). Adalimumab for Adult Patients with Rheumatological Disorders: A Review of Clinical Effectiveness.

[25] Liu F., Demosthenes P. (2022). Real-world data: A brief review of the methods, applications, challenges and opportunities. BMC Med. Res. Methodol..

[26] Mastorino L., Castelli F., Stroppiana E., Verrone A., Ortoncelli M., Susca S., Boskovic S., Passerini S.G., Macagno N., Cariti C. (2022). Risankizumab shows faster response in bio naïve than in bio-experienced psoriatic patients. J. Eur. Acad. Dermatol. Venereol..

[27] Gracia-Cazaña T., Bernal-Masferrer L., Morales-Callaghan A.M., Almenara-Blasco M., Gilaberte Y. (2023). Risankizumab for the Treatment of Moderate to Severe Psoriasis: Impact on Health-Related Quality of Life and Psychological Wellbeing. Clin. Cosmet. Investig. Dermatol..

[28] Papp K.A., Blauvelt A., Bukhalo M., Gooderham M., Krueger J.G., Lacour J.P., Menter A., Philipp S., Sofen H., Tyring S. (2017). Risankizumab versus Ustekinumab for Moderate-to-Severe Plaque Psoriasis. N. Engl. J. Med..

[29] Ruggiero A., Megna M., Fabbrocini G., Ocampo-Garza S.S. (2023). Anti-IL23 biologic therapies in the treatment of psoriasis: Real-world experience versus clinical trials data. Immunol. Res..

[30] Papp K.A., de Vente S., Zeng J., Flack M., Padilla B., Tyring S.K. (2021). Long-Term Safety and Efficacy of Risankizumab in Patients with Moderate-to-Severe Chronic Plaque Psoriasis: Results from a Phase 2 Open-Label Extension Trial. Dermatol. Ther..

[31] Megna M., Cinelli E., Gallo L., Camela E., Ruggiero A., Fabbrocini G. (2022). Risankizumab in real life: Preliminary results of efficacy and safety in psoriasis during a 16-week period. Arch. Dermatol. Res..

[32] Egeberg A., Thyssen J.P. (2023). Increased reporting of cerebrovascular accidents with use of risankizumab observed in the Food and Drug Administration Adverse Events Reporting System (FAERS). Br. J. Dermatol..

[33] Shu Y., Chen J., Ding Y., Zhang Q. (2023). Adverse events with risankizumab in the real world: Postmarketing pharmacovigilance assessment of the FDA adverse event reporting system. Front. Immunol..

[34] Summary of Product Characteristics. https://www.ema.europa.eu/en/documents/product-information/skyrizi-epar-product-information_en.pdf.

[35] Singh S., Singh S., Thangaswamy A., Thangaraju P., Varthya S.B. (2021). Efficacy and safety of Risankizumab in moderate to severe psoriasis: A systematic review and meta-analysis. Dermatol. Ther..

[36] Kristensen L.E., Keiserman M., Papp K., McCasland L., White D., Lu W., Wang Z., Soliman A.M., Eldred A., Barcomb L. (2022). Efficacy and safety of risankizumab for active psoriatic arthritis: 24-week results from the randomised, double-blind, phase 3 KEEPsAKE 1 trial. Ann. Rheum. Dis..

[37] Östör A., Van den Bosch F., Papp K., Asnal C., Blanco R., Aelion J., Alperovich G., Lu W., Wang Z., Soliman A.M. (2022). Efficacy and safety of risankizumab for active psoriatic arthritis: 24-week results from the randomised, double-blind, phase 3 KEEPsAKE 2 trial. Ann. Rheum. Dis..

[38] Östör A., Van den Bosch F., Papp K., Asnal C., Blanco R., Aelion J., Lu W., Wang Z., Soliman A.M., Eldred A. (2022). Efficacy and safety of risankizumab for active psoriatic arthritis: 52-week results from the KEEPsAKE 2 study. Rheumatology.

[39] Huang Y.W., Tsai T.F. (2020). A drug safety evaluation of risankizumab for psoriasis. Expert Opin. Drug Saf..

[40] Yan J., Smyth M.J., Teng M.W.L. (2018). Interleukin (IL)-12 and IL-23 and Their Conflicting Roles in Cancer. Cold Spring Harb. Perspect. Biol..

[41] Geller S., Xu H., Lebwohl M., Nardone B., Lacouture M.E., Kheterpal M. (2018). Malignancy Risk and Recurrence with Psoriasis and its Treatments: A Concise Update. Am. J. Clin. Dermatol..

[42] Crisafulli S., Bertino L., Fontana A., Calapai F., Ingrasciotta Y., Berretta M., Trifirò G., Guarneri C. (2021). Incidence of Skin Cancer in Patients with Chronic Inflammatory Cutaneous Diseases on Targeted Therapies: A Systematic Review and Meta-Analysis of Observational Studies. Front. Oncol..

[43] Gordon K.B., Lebwohl M., Papp K.A., Bachelez H., Wu J.J., Langley R.G., Blauvelt A., Kaplan B., Shah M., Zhao Y. (2022). Long-term safety of risankizumab from 17 clinical trials in patients with moderate-to-severe plaque psoriasis. Br. J. Dermatol..

[44] Butrón-Bris B., Daudén E., Rodríguez-Jiménez P. (2021). Psoriasis Therapy and Skin Cancer: A Review. Life.

[45] Sardella M., Lungu C. (2019). Evaluation of quantitative signal detection in EudraVigilance for orphan drugs: Possible risk of false negatives. Ther. Adv. Drug Saf..

[46] Ogdie A., Schwartzman S., Husni M.E. (2015). Recognizing and managing comorbidities in psoriatic arthritis. Curr. Opin. Rheumatol..

[47] Eder L., Harvey P., Chandran V., Rosen C.F., Dutz J., Elder J.T., Rahman P., Ritchlin C.T., Rohekar S., Hayday R. (2018). Gaps in Diagnosis and Treatment of Cardiovascular Risk Factors in Patients with Psoriatic Disease: An International Multicenter Study. J. Rheumatol..

[48] Armstrong E.J., Harskamp C.T., Armstrong A.W. (2013). Psoriasis and major adverse cardiovascular events: A systematic review and meta-analysis of observational studies. J. Am. Heart Assoc..

[49] Polachek A., Touma Z., Anderson M., Eder L. (2017). Risk of Cardiovascular Morbidity in Patients with Psoriatic Arthritis: A Meta-Analysis of Observational Studies. Arthritis Care Res..

[50] Maradit-Kremers H., Dierkhising R.A., Crowson C.S., Icen M., Ernste F.C., McEvoy M.T. (2013). Risk and predictors of cardiovascular disease in psoriasis: A population-based study. Int. J. Dermatol..

[51] Caiazzo G., Fabbrocini G., Di Caprio R., Raimondo A., Scala E., Balato N., Balato A. (2018). Psoriasis, Cardiovascular Events, and Biologics: Lights and Shadows. Front. Immunol..

[52] Roubille C., Richer V., Starnino T., McCourt C., McFarlane A., Fleming P., Siu S., Kraft J., Lynde C., Pope J. (2015). The effects of tumour necrosis factor inhibitors, methotrexate, non-steroidal anti-inflammatory drugs and corticosteroids on cardiovascular events in rheumatoid arthritis, psoriasis and psoriatic arthritis: A systematic review and meta-analysis. Ann. Rheum. Dis..

[53] Lebwohl M. (2017). Does Treatment of Psoriasis Reduce Cardiovascular Comorbidities?. J. Investig. Dermatol..

[54] Andújar I., Esplugues J.V., García-Martínez P. (2022). Looking beyond the Skin: Pathophysiology of Cardiovascular Comorbidity in Psoriasis and the Protective Role of Biologics. Pharmaceuticals.

[55] Woods R.H. (2022). Potential cerebrovascular accident signal for risankizumab: A disproportionality analysis of the FDA Adverse Event Reporting System (FAERS). Br. J. Clin. Pharmacol..

